# Results of Modified Stenströms Technique Otoplasty and Patients' Satisfaction at King Fahad Armed Forces Hospital (KFAFH)

**DOI:** 10.7759/cureus.58372

**Published:** 2024-04-16

**Authors:** Moataz Felimban, Amani J Basaeed, Rakan H Alelyani, Mansour A Dahlan, Angie M Felimban, Muath S Damanhuri, Ahmed M Alqurashi, Turki A Althobaiti, Ahmed Almenhali, Ghaidaa A Fatani, Ahmed Afandi, Rayan Hafiz, Mohammed A Althobaiti

**Affiliations:** 1 Plastic and Reconstructive Surgery, King Fahad Armed Forces Hospital, Jeddah, SAU; 2 Medicine, King Saud University Medical City, Jeddah, SAU; 3 Plastic and Reconstructive Surgery, King Saud Medical City, Riyadh, SAU; 4 Faculty of Medicine, King Abdulaziz University Hospital, Jeddah, SAU; 5 Ophthalmology, King Abdulaziz Medical City, Jeddah, SAU; 6 Plastic and Reconstructive Surgery, King Fahad Military Medical Complex, Jeddah, SAU; 7 ENT, King Faisal Medical Complex, Taif, SAU; 8 Otolaryngology - Head and Neck Surgery, Alhada Military Hospital, Taif, SAU; 9 Medicine, King Abdulaziz University Faculty of Medicine, Jeddah, SAU; 10 Surgery, King Fahad Armed Forces Hospital, Jeddah, SAU; 11 Plastic and Reconstructive Surgery, King Faisal Medical Complex, Taif, SAU

**Keywords:** plastic surgery, external ear deformity, bat ears, surgical technique guide, prominent ear

## Abstract

Background

Globally, the prevalence of protruding ears is relatively frequent. Ear deformities manifest due to underdevelopment of the antihelical fold, conchal hypertrophy, and/or an obtuse conchoscaphal angle. The availability of multiple approaches proves that there isn't a single optimal accepted procedure. The Modified Stenström otoplasty technique supports the surgeon in the management of underdeveloped antihelix fold, conchal hypertrophy, and obtuse conchoscaphal angle among other deformities. We are the first to evaluate the clinical effects and measure the satisfaction rate post-otoplasty using the modified Stenström technique with a case series study.

Methods

Six patients were included in the study with a total of 12 ears operated on between February 2021 and July 2022. Utilizing the modified Stenström technique for bilateral protruding ears. All patients had six postoperative follow-up visits with fixed intervals; one week, three weeks, six weeks, three months, six months, and one year. During their one-year postoperative follow-up appointment, all patients completed the satisfaction survey questions.

Results

Six individuals were studied, three males and three females with a mean age of 23.1 (range, 7-53 years old). There were no complications or recurrences observed. Based on the responses we collected, all patients reported a high satisfaction rate at one-year postoperative follow-up.

Conclusion

The modified Stenström technique yields good naturally appearing ears. It is an easy and safe technique to apply. It has a short recovery period, and no hospital stay is required. All contribute to a high satisfaction rate among studied patients.

## Introduction

One of the most prevalent congenital deformities in the head and neck is prominent ears [1], which can occur anywhere between 0.5% and 15% of the time, with a Caucasian prevalence of 5% [2]. This deformity occurs either as a result of embryological malformation during the fifth to ninth weeks of gestation or due to an external compression in early fetal development resulting in distorted ear growth [3]. This malformation manifests as one or more of the following: underdevelopment of the antihelical fold, conchal hypertrophy, or an obtuse conchoscaphal angle [4]. Prominent ear deformity is evidenced to be associated with great psychological distress, low self-confidence, negative body image, and difficulties with social interactions [5]. For the correction of ear protrusion deformities, many surgical techniques have been used. One of the most used procedures is the Stenström technique, which relies on the weakening of the cartilage and recovery in the desired anatomic position, enhancing or creating the antihelical fold [4]. This consists of an anti-helix plication by anterior cartilage rasping and elliptical posterior skin excision without sutures [6,7]. However, this technique is limited to the correction of an underdeveloped antihelical fold only. Surgical modification and refinement of the Stenström technique have been proposed in the event of conchal hypertrophy and obtuse concoscaphal angle [4]. Resection of a small part of the concha cymba and scapha is performed via a posterior approach in case of conchal hypertrophy [4], with the rasp being introduced via an anterior approach and concealed beneath the upper plication of the helix. In the case of an obtuse conchoscaphal angle, the posterior auricular muscle is resected, followed by burying of the concha by suture to the mastoid periosteum [8]. This modification was first implanted in France with successful results at one-year post-operation [4].

In this study, we aim to assess patients' satisfaction with this modification and refinement of the Stenström technique.

## Materials and methods

**Figure 1:**
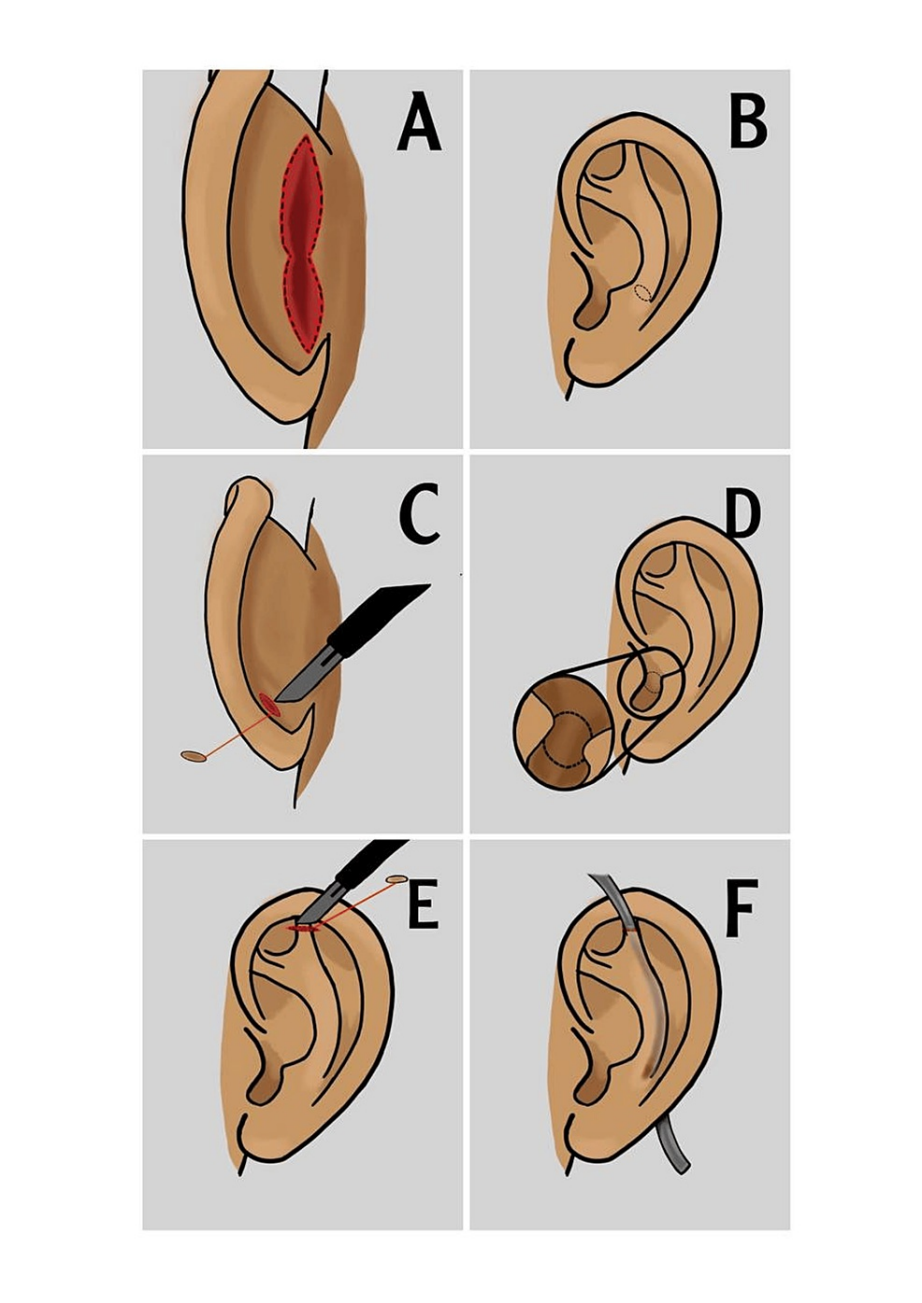
Operative steps in modified Stenströmtechnique for otoplasty This illustration was produced and drawn by co-author Mansour A. Dahlan.

This is a case series study involving six patients during the period February 2021 to July 2022 at King Fahad Armed Force Hospital, Jeddah, Saudi Arabia. All patients met the inclusion criteria, in which there was a prominent ear deformity, without other auricular malformation, no previous auricular surgeries, and were treated by the same surgical team using a modified Stenström technique (Figure 1 and Table 1) with a follow-up period of not less than six months.

**Table 1:** Operative steps in modified Stenström technique for otoplasty

1- Marking of the appropriate desired position of the antihelix on the anterior surface of the ear.
2- An hourglass 5-6 cm longitudinal incision is made on the posterior surface of the ear, about 1-2 cm from the sulcus outwardly (Figure 1 - sketch A).
3- The tail of the marking of the antihelix is dissected through the posterior incision, and a small 0.3-0.5 cm square part of the cartilage at the caudal part of the antihelix is excised in a longitudinal fashion (Figure 1 - sketches B and C). (This step prevents lobule protrusion after antihelix plication in later steps.)
4- In the case of conchal hypertrophy: based on marking on the anterior skin surface resection of a small round part of the concha cymba cartilage is carried out through the posterior incision, with a careful technique to not violate the skin surface anteriorly (Figure 1 - sketch D).
5- In the case of an obtuse conchoscaphal angle, dissection is carried out to reach and incise the posterior auricular muscle via the posterior longitudinal incision.
6- Anteriorly a small incision is made along the assumed most superior crus point of the marked antihelix.
7- Through this incision, a small oval shape of the cartilage is excised anteriorly; this step creates a tunnel gate for rasping of the cartilage and prevents lobule protrusion after plication of the antihelix, respectively (Figure 1 - sketch E).
8- Through the anterior superior crus incision on the anterior surface, a Stenström rasp is introduced and weakening of the cartilage along the assumed antihelix is performed. The rasp is advanced to reach the caudal point of the antihelix incision posteriorly (Figure 1- sketch F).
9- All cartilage fragments are washed out with normal saline flushing through the anterior incision with a cannula.
10- Posteriorly, utilizing the Furnas technique a conchal setback suture is placed attaching the concha to the mastoid fascia with a 3/0 Monocryl suture and secured with a mosquito without closing the knot until the final simulation of the desired anatomy of the antihelix is achieved.
11- Utilizing the Mustardé technique, an antihelix plication suture is placed with 3/0 Monocryl and secured with a mosquito. The number of sutures required varies from 2 to 3 to sufficiently restore the antihelical rim.
12- After the desired contour of the antihelix is achieved with the Mustardé technique, completing the Furnas suture posteriorly is carried out first then the Mustardé suture.
13- Posterior skin incision is closed with 4/0 Monocryl.
14- Dressing is done with Vaseline gauze and crepe bandage with light pressure. The first dressing session is after 24 hours, and then performed every 48-72 hours subsequently following the same dressing protocol for a total duration of 14 days.

They were followed up at a fixed period with a total of six visits in one week, three weeks, six weeks, three months, six months, and one-year post-operation.

All patients received a satisfaction survey, one year after the surgery, consisting of three questions. Overall patients’ satisfaction with the surgery outcomes and the appearance of the ear were graded on a scale as the following: very happy=5, happy=4, neutral=3, unhappy=2, and sad=1. Symmetry of the ear scale had the following grades: excellent=5, good=4, fair=3, bad=2, poor=1. McDowell and Wright's goals were applied to the postoperative assessment [9,10].

Ethical statement

This study was approved by the research ethics committee at King Fahad Armed Forced Hospital under reference number 559. There was a requirement for formal informed consent that was taken from all involved participants in this study; moreover, all medical history and clinical details, including the patient’s information, were anonymized to guarantee that only anonymous data were analyzed.

## Results

The study included six patients with 12 ears in total. All patients underwent bilateral otoplasties with the modified Stenström technique. These six patients, who ranged in age from 7 to 53 years (mean age, 23.1 years), included 3 males and 3 females. Six postoperative follow-up visits were conducted over a year. During the follow-up period, no postoperative complications were recorded. None of the patients experienced recurrent protrusions or telephone ear deformities at the end of the one-year follow-up visits.

The patient characteristics and satisfaction survey are shown in Table 2.

**Table 2:** Patient demographics and satisfaction survey at the one-year follow-up

Patient	Age	Gender	Overall satisfaction with your surgery	Overall satisfaction with the appearance of your ear	What do you think about your ears' symmetry?	Follow-up
1	11	Girl	Happy	Happy	Very good	6 visits for 12 months
2	10	Girl	Very happy	Happy	Excellent	6 visits for 12 months
3	7	Boy	Very happy	Happy	Excellent	6 visits for 12 months
4	40	Male	Happy	Happy	Excellent	6 visits for 12 months
5	53	Female	Very happy	Very happy	Excellent	6 visits for 12 months
6	18	Male	Very happy	Happy	Excellent	6 visits for 12 months

Case 1

**Figure 2:**
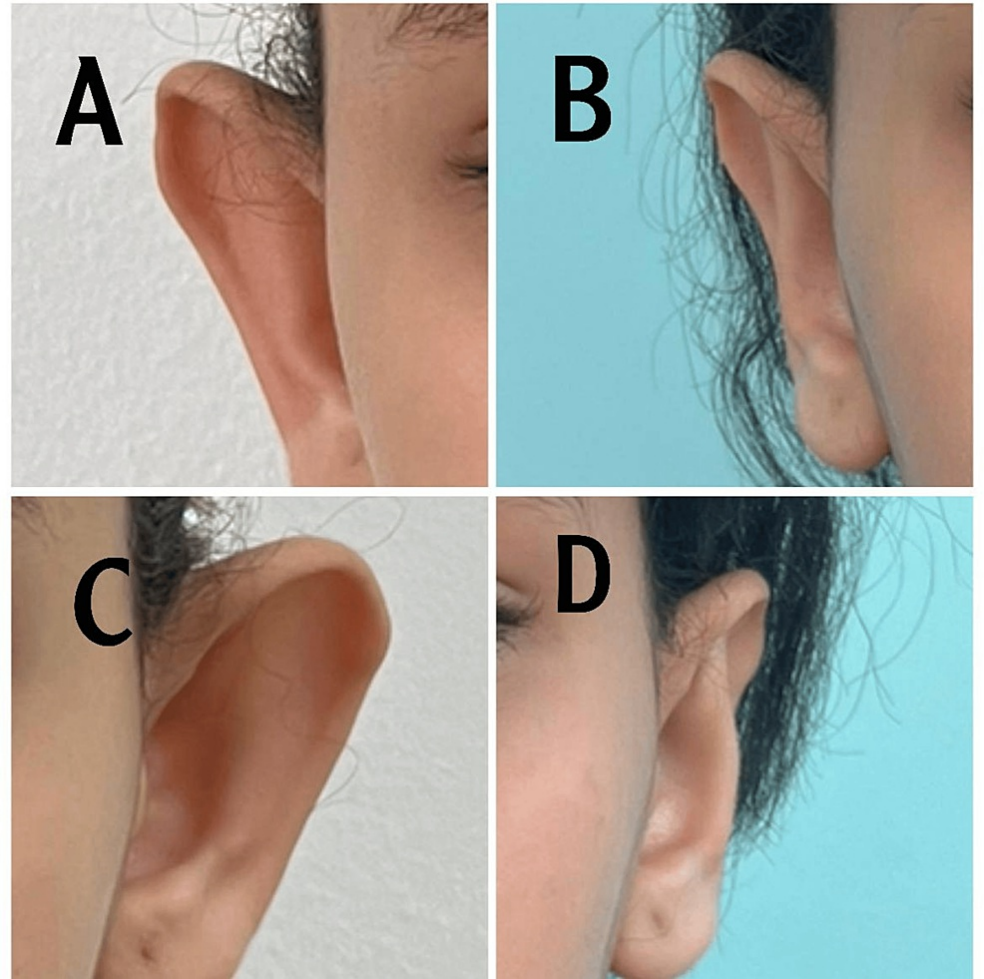
A. Preoperative right ear, B. Postoperative right ear, C. Preoperative left ear, D. postoperative left ear Case 1

This is a case of an 11-year-old girl. The patient has an obtuse conchomastoid angle measured at 120 with an auriculocephalic angle of 83. This widened angle contributed most to the prominent ear bilaterally. The absence of an anti-helix along with cupping of the conchal cartilage due to the excess was noted (Figure 2). Postoperative pictures were taken six months after the operation. Slight asymmetry is seen preoperative of the left ear, which is more protruded than the right ear. We managed to correct them as best as possible; postoperatively, there was slight asymmetry as expected.

Case 2

**Figure 3:**
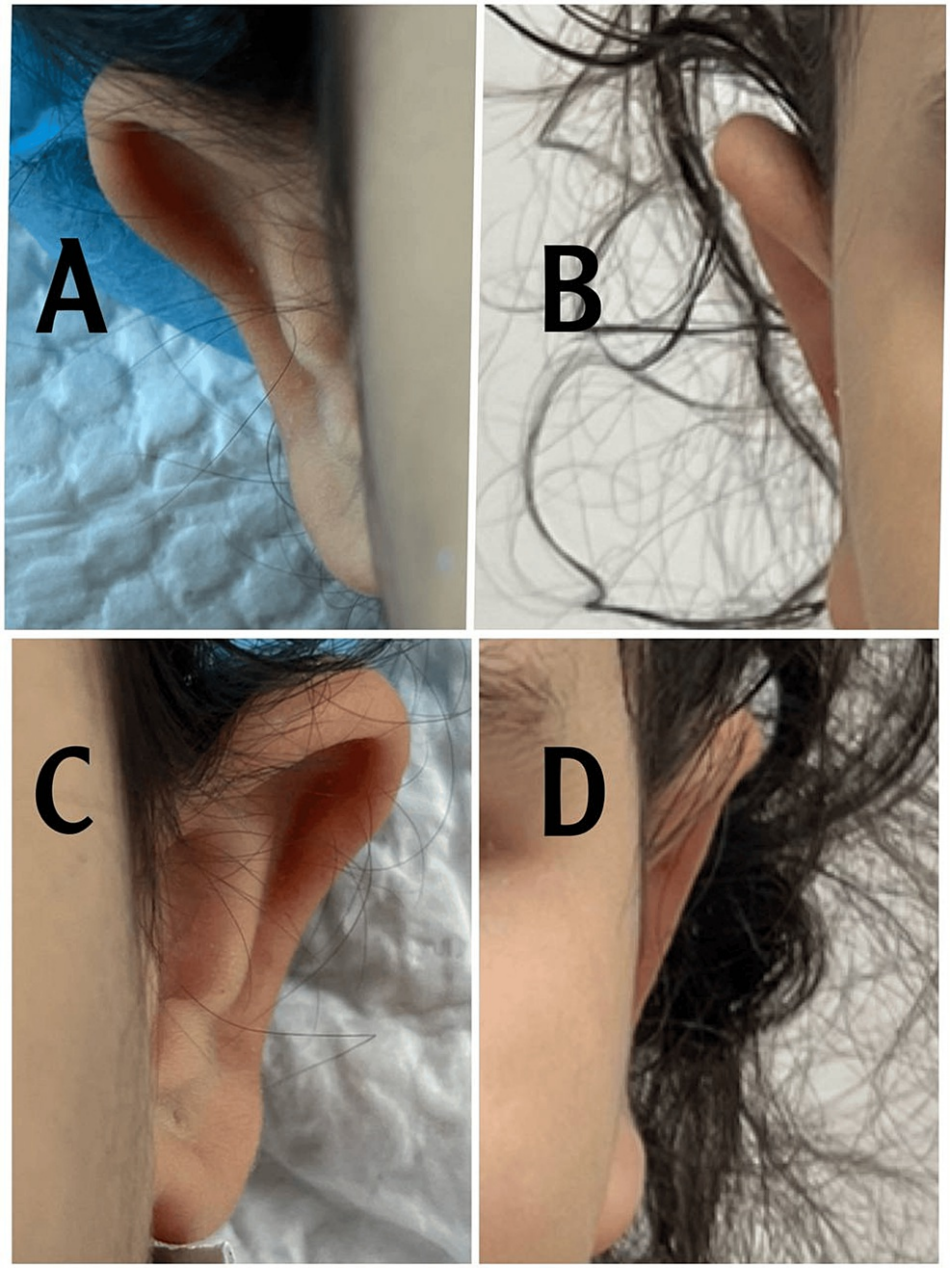
A. Preoperative right ear, B. Postoperative right ear, C. Preoperative left ear, and D. Postoperative left ear Case 2

This is a case of a 10-year-old girl. The patients' measured auriculocephalic angle was 80, with a poorly defined anti-helix, along with excess conchal cartilage and a wide conchomastoid angle of 105 (Figure 3). Postoperative pictures were taken three weeks after the operation. An over-projected upper part of the ear was noticed preoperative and corrected to its maximum intraoperative; the appearance of telephone ear deformity in the postoperative pictures could be due to an inappropriate postop photograph angle.

Case 3

**Figure 4:**
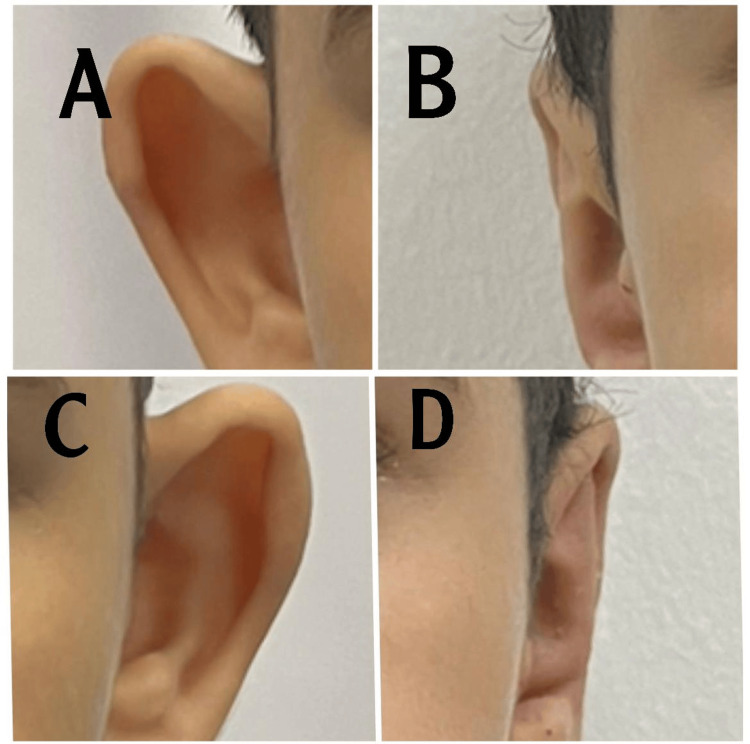
A. Preoperative right ear, B. Postoperative right ear, C. Preoperative left ear, and D. Postoperative left ear Case 3

This is a case of a seven-year-old boy. He presented with bilateral ear and had an estimated measure of auriculocephalic angle of 75° corresponding to the obtuse conchoscaphal angle of about 120° and there was also an underdeveloped anti-helix with mild excess conchal cartilage resulting in the prominence of the ear (Figure 4). Postoperative pictures were taken three weeks after the operation. In this case, we over-corrected the ears due to severe preoperative bilateral ear protrusion from the scalp, to overcome any unpleasant protrusion postoperative.

Case 4

**Figure 5:**
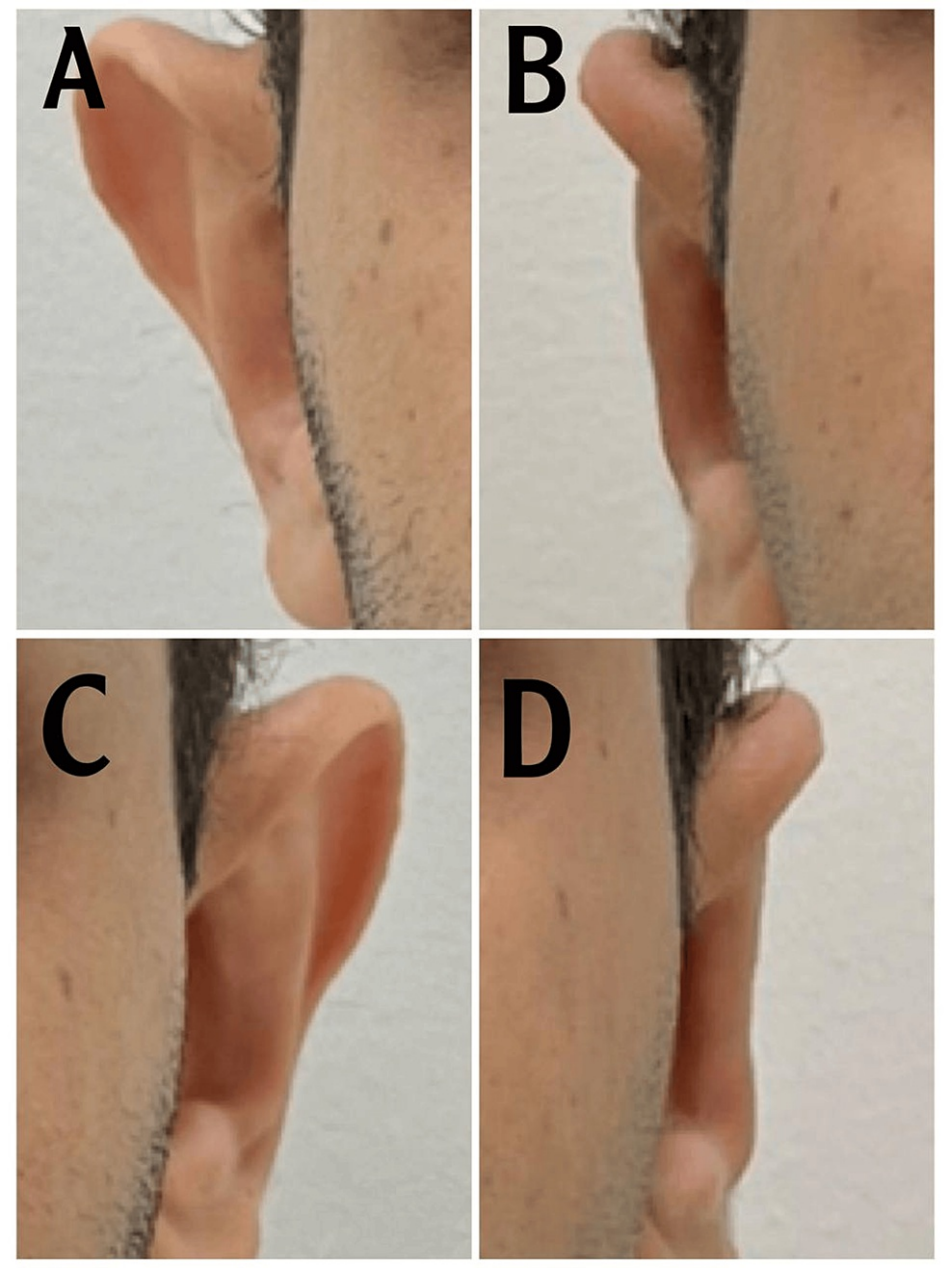
A. Preoperative right ear, B. Postoperative right ear, C. Preoperative left ear, and D. Postoperative left ear Case 4

This is a case of a 40-year-old man. In this case, the prominent ear is mostly a result of an underdeveloped anti-helix, which widened the conchoscaphal angle to about 128° with an auriculocephalic angle of 82° (Figure 5). Postoperative pictures were taken three months after the operation.

Case 5

**Figure 6:**
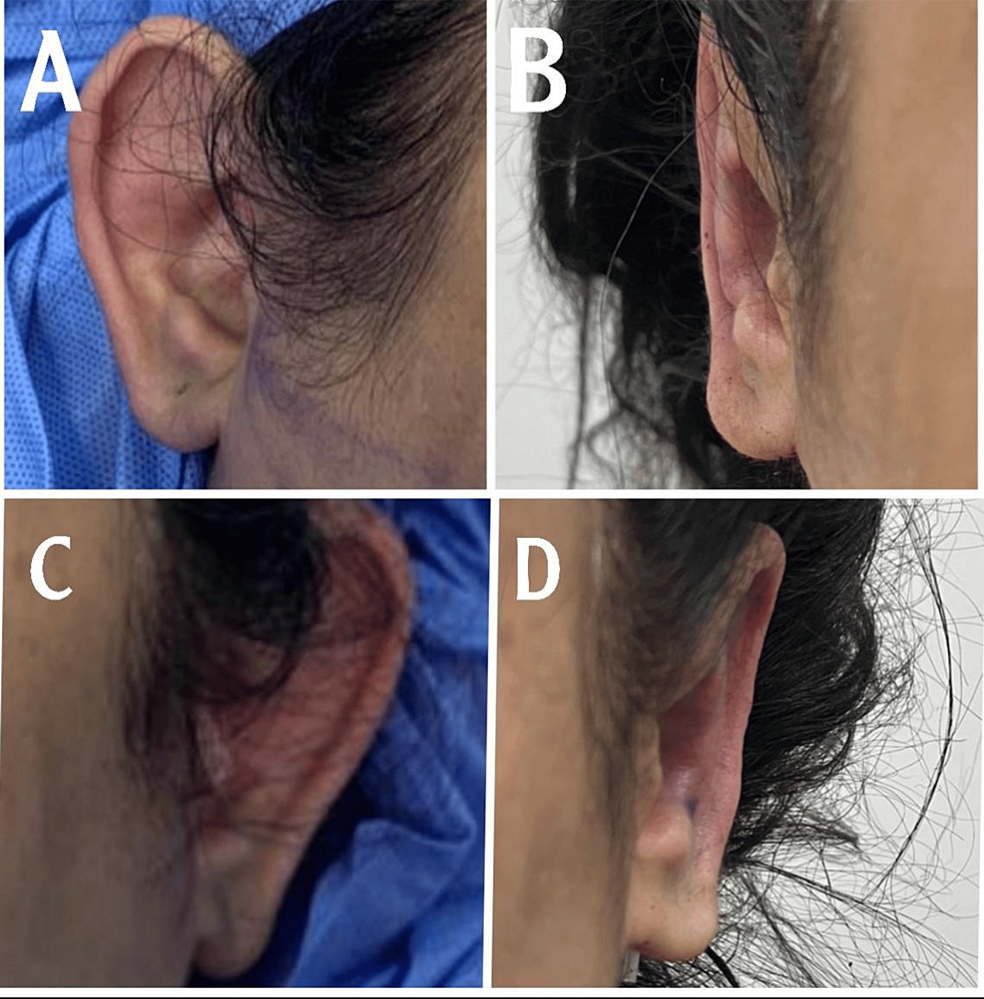
A. Preoperative right ear, B. Postoperative right ear, C. Preoperative left ear, and D. Postoperative left ear Case 5

This case is a 53-year-old female who presented with an obtuse conchomastoid angle of more than 110° and an auriculocephalic angle of 86°. An underdeveloped anti-helix along with excess conchal cartilage is noted preoperatively (Figure 6). Postoperative pictures were taken three months after the operation.

Case 6

**Figure 7:**
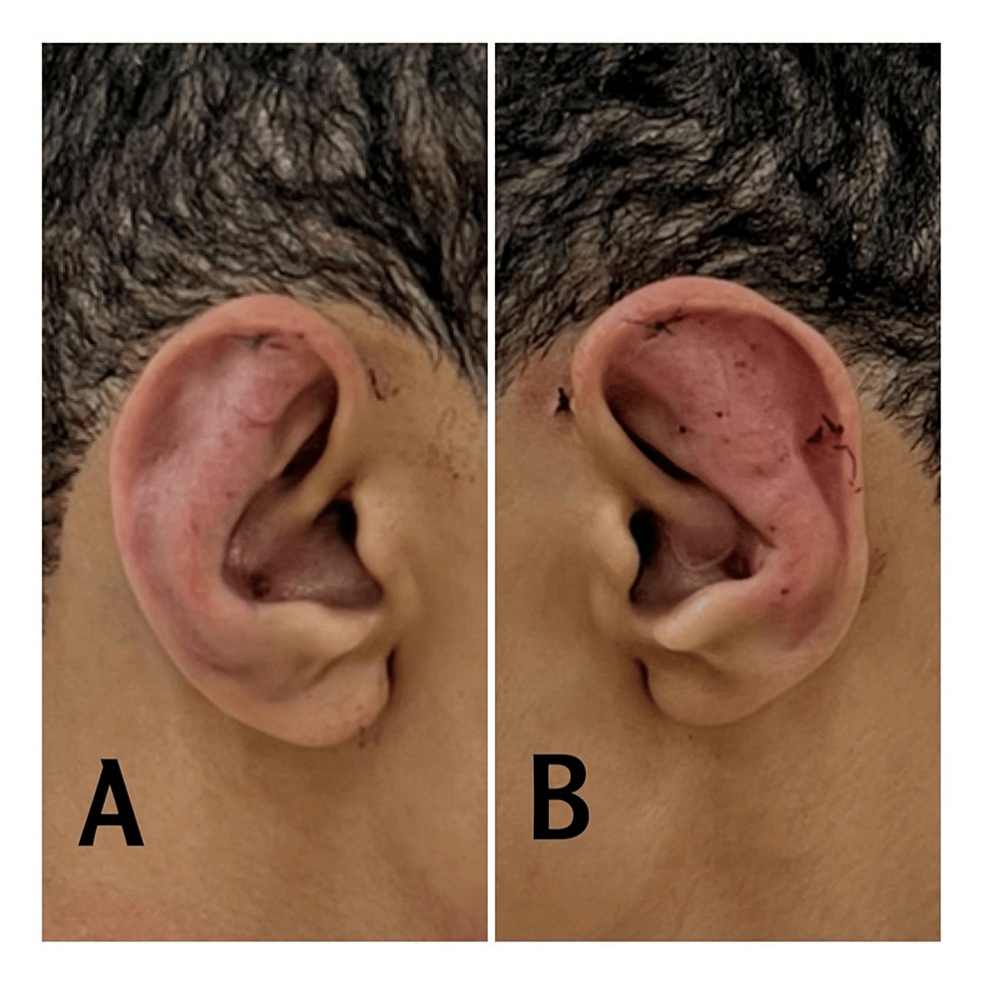
A. Postoperative right ear and B. Postoperative left ear Case 6 Preoperative images were obtained with very low quality, so they were discarded.

This is a case of an 18-year-old male. This patient presented with a large, outwardly rotated conchal cartilage and with a measured auriculocephalic angle of 89. An underdeveloped anti-helix and obtuse conchomastoid angle were also present (Figure 7). Postoperative pictures were taken one week after the operation. This picture was taken on day 6 postoperative, there is some edema postoperative, and signs of inflammation due to compression bandages.

We observed in the clinic that every patient was highly satisfied with the final operative outcome (Figures 2-7). When it came to overall satisfaction with the ears' appearance and their surgical experience, we found that every patient was either very happy or happy. All patients and parents rated the symmetry as being very good to excellent. During each follow-up visit, a measurement of the distance from the mastoid to the helix distance was checked and confirmed to be within the normal range of 2-2.5 cm.

## Discussion

A prominent ear is a common deformity, and despite not posing a physiological disability, it has a significant impact on social integration and psychological well-being, particularly in younger individuals. As such, surgery is typically recommended at a young age [11,12]. Age at the time of operation varies in the literature because aesthetic complaints are the main reason for surgery. Based on the available literature, between 35% and 70% of patients are female, and this number rises beyond the age of 20 [13]. The patients in our study had a mean age of 23.1 years. Three of them were female, and three were male.

To address this deformity, numerous surgical techniques have been developed since Dieffenbach performed the surgery for the first time in 1845. For the treatment of prominent ears, over 200 techniques are currently in use [14]. They can be classified as cartilage-sparing or cartilage-cutting techniques. These include percutaneous, incisionless, endoscopic, cartilage-sparing, and splitting techniques. Nowadays, the Stenström technique is popular in France and the rest of Europe, while the Mustardé technique is popular in England and North America [15].

The fact that different methods are available implies that there isn't one best or universally acknowledged process [16,17]. Our modified Stenström otoplasty technique aids the surgeon when treating obtuse concho-scaphal angle and conchal hypertrophy. They can be used to correct the most common ear defects of prominent ears and are reliable and simple to execute.

The modified shape of cartilage healing is the foundation of the traditional Stenström technique. To prevent the sutures from possibly extruding, no permanent sutures are used to create the antihelix plication. There are reports of this extrusion in 12.5% of the cases [18]. A previous study by Sadhra et al. demonstrated that the incidence of hematomas can reach up to 3.8%, postoperative infections 1.3%, wound complications 5.1%, suture problems up to 2.6%, and revision surgery up to 7.7% based on an analysis of data from 3,493 patients in the literature who underwent otoplasty for prominent ear [19]. Another study conducted by Punj et al. reported that postoperative complications (bleeding, wound infection, recurrence) occurred in 2.2%, 0.9%, and 10% of cases, respectively, using the Chongchet or Mustardé technique [20].

We have modified the Stenström technique to correct for conchal hypertrophy and obtuse concho-scaphal angle, among other deformations. Moreover, different studies showed a significant percentage of complications with other techniques. Smittenberg et al., Maricevitch et al., and Valentines et al. reported the rate of complication as 20%, 12.8%, and 10%, respectively [21-23].

Our method, the modified Stenström technique, is simple to apply and safe to use. In this case series study, there were no postoperative complications in our patients following the procedure. and no hospital stay was required. The overall results were good and natural. All six patients expressed great happiness and satisfaction with the results.

The pros of our modification are that it is easy to introduce the rasp with more accurate cartilage shaping, and when we irrigate the area that has been rasped, we are sure that there will be no more cartilage particles left in the area because they drain from the inferior part. The cons are that a learning curve is needed to master the modifications, anterior skin perforation could be expected if the rasping process is done blindly without taking care of the rasping technique, and the surgery needs more time.

To conclude, there is no specific technique for otoplasty that is superior to others. Yet, there are multiple techniques each addressing a specific defect that causes protuberant ears. We have found that this modified technique is suitable to address the common defects that are known to cause protuberant ear deformity as mentioned in the article. Thus, following this technique has been shown to correct all three causes of protuberant ears. Further studies with a bigger sample size will better demonstrate the outcomes of the technique. In terms of corrected angles, it was documented to elaborate on the severity of the defect so the reader can have a better perspective of the case. However, we preferred to view the outcomes of the procedure through the patient’s satisfaction and measured the mastoid to helix distance to be sufficient and accepted when within the normal range of 2-2.5 cm.

Limitations

This case series looked into satisfactory outcomes post otoplasty using the new modified Sternströms technique with six cases. One limitation was the small number of cases studied and the duration of follow-up. A longer follow-up period would be more informative to account for any late recurrence. Moreover, there was a reliance on self-reported satisfaction and a lack of all preoperative and postoperative pictures with all views (anterior, posterior, and lateral) at specific time periods.

## Conclusions

For the correction of prominent ears, the modified Stenström technique, which treats obtuse concho-scaphal angle and conchal hypertrophy is safe, effective, has a short recovery period, and yields good long-term outcomes. In this case study there were no postoperative complications, recurrences, or telephone ear abnormalities recorded. Overall, patients reported a high satisfaction rate with the surgery, general appearance, and symmetry of the ear. We urge that future studies be undertaken prospectively, with bigger sample sizes and longer follow-up periods, to ensure the validity of the results.
